# RF shimming strategy for an open 60‐channel RF transmit 7T MRI head coil for routine use on the single transmit mode

**DOI:** 10.1002/mrm.30563

**Published:** 2025-05-20

**Authors:** Andrea N. Sajewski, Tales Santini, Anthony DeFranco, Jacob Berardinelli, Hecheng Jin, Jinghang Li, Cong Chu, Jeremy J. Berardo, Tamer S. Ibrahim

**Affiliations:** ^1^ Department of Bioengineering University of Pittsburgh Pittsburgh Pennsylvania USA; ^2^ Department of Psychiatry University of Pittsburgh Pittsburgh Pennsylvania USA

**Keywords:** 7T RF coils, B_1_ field, RF shimming, SAR, Tic Tac Toe Design

## Abstract

**Purpose:**

To develop an radiofrequency (RF) shimming approach for operating the 2nd Generation Tic Tac Toe RF coil system (60 transmit channels integrated with 32‐channel receive insert) for routine use in 7T neuro MRI on the single transmit mode.

**Methods:**

RF simulations were performed and used to develop non‐subject‐specific RF shim cases over three anatomically detailed head models: adult male, adult female, and child female. Multi‐ROI shimming strategies were developed and implemented. $B_{1}^{+}$ maps and in vivo images were acquired on the single transmit mode of a 7T scanner using the RF shim cases derived from the computer simulations.

**Results:**

The availability of 60 transmit channels enables more control over $B_{1}^{+}$ efficiency, specific absorption rate (SAR) efficiency, and $B_{1}^{+}$ homogeneity using RF shimming. On the single transmit mode, the 2nd generation Tic Tac Toe RF coil system consistently provides homogeneous $B_{1}^{+}$ field distribution with extended coverage into the temporal lobes, cerebellum, reaching all the way to C5–C6. Safe levels of SAR are also achieved.

**Conclusion:**

By using a non‐subject specific RF shimming approach derived from computer simulations, the 2nd generation Tic Tac Toe RF coil system allows for robust, routine neuroimaging (>1750 in vivo scanning sessions over the past 28 months) at 7T in single transmit mode.

## 1 INTRODUCTION

Ultra‐high field (UHF) human MRI, designated as having a primary magnetic field (B_0_) strength of 7T or higher, has shown clinical utility in neuroimaging and musculoskeletal imaging,^1^, ^2^ offering improvements in signal‐to‐noise ratio,^3^, ^4^ contrast enhancement,^5^ higher spatial and spectral resolution, and improved BOLD sensitivity.^6^, ^7^ With United States Food and Drug Administration (FDA) clearance for multiple vendors in recent years,^8^, ^9^ 7T sites have been increasing for clinical and research use.

Despite its advantages, UHF MRI still poses technical challenges because of the increased operational frequency and thus reduced wavelength (˜13 cm for in vivo proton neuroimaging at 7T). This shortened wavelength leads to spatial inhomogeneity of the radiofrequency (RF) fields^3^, ^10^: inhomogeneity in the transmitted magnetic field ($B_{1}^{+}$) may lead to signal dropout in the MR images, while electric field inhomogeneity may lead to increased localized tissue heating quantified by specific absorption rate (SAR). Additionally, UHF MRI systems lack a built‐in body transmit coil, requiring custom or commercial transmit coils for neuroimaging. The scarcity of commercial systems and the challenge of achieving consistent whole‐brain homogenous excitation has driven research groups to develop custom transmit coils.^11^, ^12^ Various UHF coil designs have been introduced beyond the traditional birdcage design, including loops,^13^, ^14^, ^15^ dipoles,^11^, ^16^ microstrips,^17^ and hybrid systems.^18^

One approach to address spatial inhomogeneity is to utilize a multichannel transmit system, in which each transmit channel can be driven independently and the resultant RF fields can be combined with superposition.^19^ RF shimming, the process of optimizing the amplitudes and phases of a multichannel transmit system, is used to help mitigate the inhomogeneity of the electric and magnetic fields.^20^, ^21^ This can be done on a per‐subject basis using a parallel transmission (pTx) system, which has been able to provide more homogeneous $B_{1}^{+}$ fields,^22^ but has longer setup times and was only recently FDA cleared for clinical use.^23^ For sites with existing 7T systems, the single‐transmit (sTx) mode of the scanner is often used, requiring non‐subject‐specific RF transmit systems. However, sTx coils based on the traditional quadrature birdcage design, which are widely used, usually struggle to provide sufficient $B_{1}^{+}$ intensity to the entire brain, especially in the temporal lobes and cerebellum.^24^, ^25^ The use of dielectric pads could potentially improve the signal in these lower brain regions^24^, ^25^; but this comes at the cost of SAR changes and/or inducing field inhomogeneities in other regions.^25^, ^26^ Furthermore, to improve $B_{1}^{+}$ efficiency for UHF MRI, many coil designs are fully covered in front of the face and visual field, not only creating challenges with patient comfort and usability, but also limiting the viewing of visual stimuli as is often required for functional MRI experiments.

The 1st Generation Tic Tac Toe RF coil system, established previously,^27^, ^28^, ^29^ demonstrated the ability to provide a homogeneous and load‐insensitive $B_{1}^{+}$ field for neuro MRI at 7T in sTx mode with 16 combined transmit channels.^30^, ^31^, ^32^ In this work, we examine RF shimming on the next‐generation Tic Tac Toe transmit coil design.^33^ By increasing the number of transmit channels, the degrees of freedom in RF shimming increases, allowing for potential improvements in homogeneity.^20^, ^34^, ^35^ We analyzed non‐subject‐specific RF shimming strategies for this multi‐channel transmit array for use with sTx systems. We performed finite difference time domain (FDTD) simulations and numerical optimizations over three anatomically detailed head models to develop three different RF shim cases, each of which was experimentally implemented and tested in vivo on 7T. By shimming over the three head models simultaneously, we were able to achieve a robust coil operation in sTx mode for routine neuro imaging at 7T.

## 2 METHODS

### 2.1 Tic Tac Toe design

**FIGURE 1:**
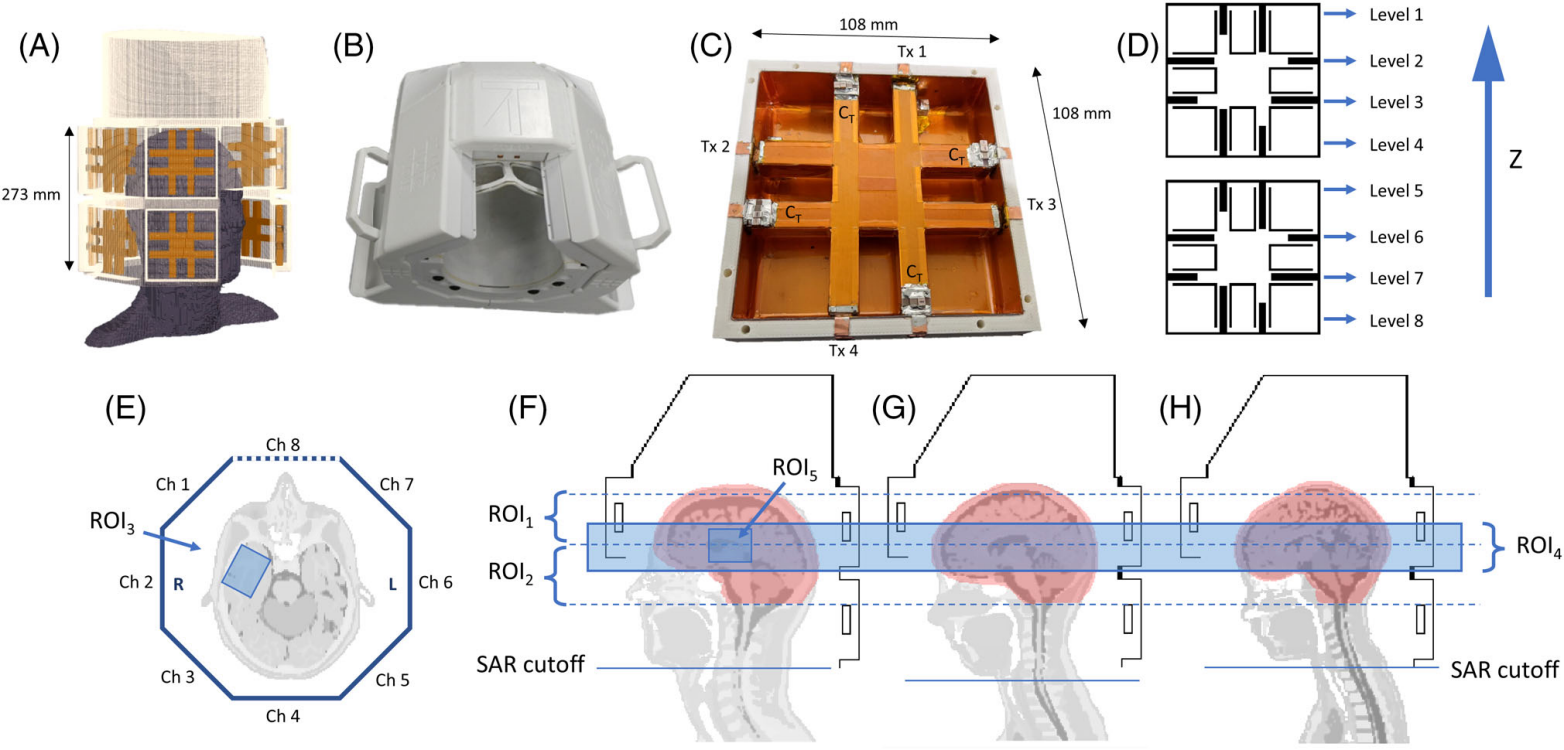
Second generation Tic Tac Toe coil design: (A) 3D model of the FDTD grid. (B) Photo of the coil with cover. (C) Single TTT panel, with mapping of transmission lines (Tx 1–Tx 4), and tuning capacitors (*C*_T_). (D) Cross section of the FDTD grid through the center of the panel, showing the rod positionings and the mapping of the Z‐levels of the coil, where level 1 is highest in the Z direction and at the top of the head. (E) Mapping of channels within each level around the coil. The top row of the coil has eight channels per level while the bottom row has seven, since it omits the panel in front of the face. (F–H) Cross‐section of FDTD grid showing the positions of (F) Duke, (G) Ella, and (H) Billie in the transmit coil. Dotted lines indicate the cutoffs for ROIs 1 and 2 used in RF shimming. The blue overlays in (E) and (F–H) represent the additional ROIs (3, 4, and 5) used in the optimization of RF shim case 3. The mask used in RF shimming and to calculate $B_{1}^{+}$ statistics is shown in (F–H) overlaid in red on each model. The lower bound for SAR calculations is displayed as a solid line below the chin for each head model.

The Tic Tac Toe (TTT) panel consists of eight transmission lines connected to each other, with half used as RF inputs and half for frequency tuning.^28^, ^29^, ^30^, ^31^, ^32^ Solid copper rods are inserted into each leg of the dielectric TTT structure to create the coaxial transmission line and can be adjusted for tuning and matching; capacitors are used for further tuning of the coil (Figure 1C,D). In this design, each TTT panel is 98 mm × 98 mm × 23 mm for the inner copper shield. The distance between the strut and the shield was optimized for homogeneity and SAR reduction.^36^ A total of 15 TTT panels are arranged in a two‐row octagonal array around the head (Figure 1A,B), providing a total of 60 transmit channels. The coil is open in front of the face from eye level to promote patient comfort and to allow easy access to fMRI projection. The shielding extends 144 mm beyond the top row of the coil to accommodate the preamplifier boards for the receive insert and is slanted to increase the field of view for fMRI stimulus projection.

### 2.2 RF simulation

The transmit coil geometry was generated using in‐house code written in Python3.^37^ The RF fields ($B_{1}^{+}$ and electric fields) produced by each transmit channel were simulated individually using in‐house developed FDTD software with dedicated transmission line and capacitor models.^27^, ^38^ Dimensions were 256 × 256 × 340 Yee cells, including 32 perfect matching layers on the bottom of the model (−Z), 8 at the top (+Z), and 24 on all sides (X and Y). The Yee cell size was 1.59 mm isotropic. Time resolution was ˜3 picoseconds, and 100 000‐time steps were used to reach steady state. For simplicity, the receive coil was omitted from the simulations, as previous work has shown that the receive coil has minimal impact on the $B_{1}^{+}$ fields for TTT designs (an average of 1.62% change in mean $B_{1}^{+}$ per channel).^39^ These methods have been previously validated to provide an accurate estimation of the $B_{1}^{+}$ and electric fields.^21^, ^29^, ^30^, ^32^, ^40^, ^41^

Anatomically detailed head models (Duke, Ella, and Billie, IT'IS Foundation Virtual Family^42^), cropped at the shoulders, were used in the simulations. The Duke model represents an adult male, age 34, weighing 70.2 kg, Ella represents an adult female, age 26, weighing 57.3 kg, and Billie represents a child female, age 11, weighing 34 kg. To compare brain size, intracranial volume (ICV) for each of the models is as follows: Duke, 1.61 L; Ella, 1.57 L; and Billie, 1.41 L. The models were placed within the coil considering the shoulders and as far back as possible toward the top of the receive insert. Their positions within the Tx coil are displayed in Figure 1F–H.

### 2.3 Optimization

Non‐subject‐specific RF shimming was performed using MATLAB (The MathWorks) optimization functions (fmincon, GlobalSearch, interior point) to modify the amplitudes and phases of the input pulse on each channel, based on methods previously described.^30^ The resolution of the $B_{1}^{+}$ fields was reduced to 3.18 mm isotropic to increase the speed of the computation. For each head model, the region of interest (ROI) used was the whole head from the bottom of the cerebellum to the top of the brain, excluding the nasal cavities and ears, as shown as an overlay in Figure 1F–H. The mean $B_{1}^{+}$ field in the whole‐head ROIs was constrained to produce a flip angle of at least 180° per 548 V at the coil plug (achievable with our standard 8 kW power amplifier) for 1 ms square pulse to ensure adequate $B_{1}^{+}$ fields across the entire brain.

For the first RF shim case (Case 1), in order to achieve a simple, straightforward implementation, amplitudes were restricted to implementable values such that each Z‐level of the coil (Figure 1D) would be driven through the same splitter (i.e., the channels belonging to a Z‐level would have the same amplitude).^30^ Coefficient of variation (CV = standard deviation/mean) of $B_{1}^{+}$ was used as the initial cost function for preliminary optimizations on the Duke model starting from the 13 819 possible amplitudes with a maximum of three levels of splitters, using 1‐, 2‐, 3‐, or 4‐way splitters.

**FIGURE 2:**
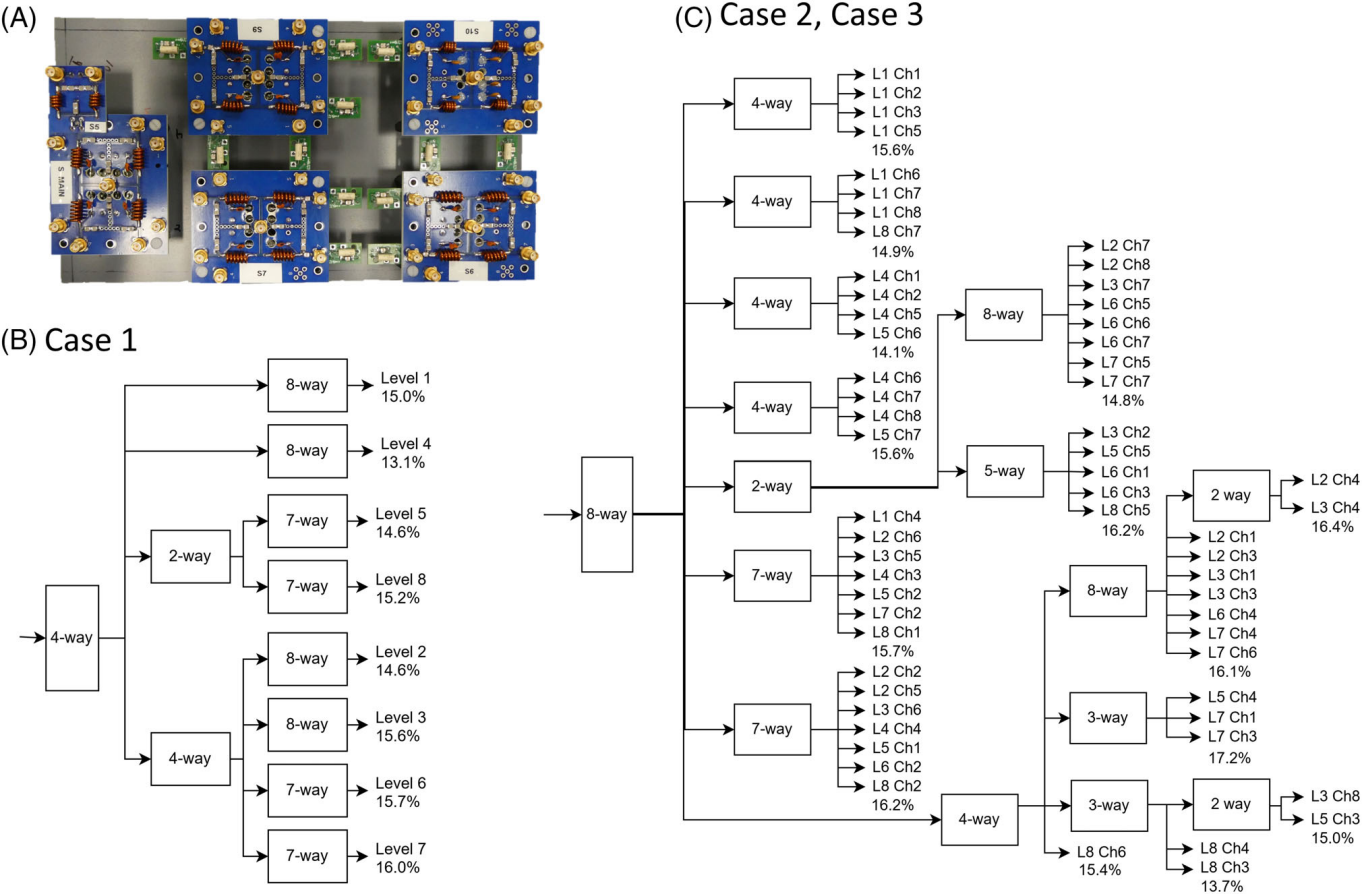
Splitter configuration for the optimized shim cases. (A) Photo of some of the boards used in the physical splitter assembly in cases 2 and 3. (B) Splitter configuration for RF shim case 1, where all seven or eight channels within a Z‐level are held at the same amplitude and phase‐only shimming is performed. (C) Splitter configuration for RF shim cases 2 and 3, which use amplitude and phase shimming. Below each set of levels or channels originating from a single splitter are the average losses from those channels, measured from the coil plug to the end of the cables before the coil ports. Level and channel mappings can be found in Figure 1D,E.

From these preliminary shim cases, seven were picked with low CV and used as a starting point for further optimizations using multiple head models. GlobalSearch used 40 initial trial points. Phase‐only RF shimming was performed using a cost function of

(1) $$\begin{array}{c} \text{Cost}=\frac{{\mathrm{CV}}_{\text{Duke}}}{\left(a*\min_{1}+b*\min_{2}\right)}\;\\ \text{Constraint}=\text{mean}>180{}^{\circ}/548V \end{array}$$

where *CV*_Duke_ is the CV of $B_{1}^{+}$ in the Duke ROI, and *min*_*1*_ and *min*_*2*_ are the $B_{1}^{+}$ minimums over all three head models (Duke, Ella, and Billie) in the top half of the ROI (between the top dotted lines in Figure 1F–H, labeled as ROI_1_) and bottom half of the ROI (between the bottom dotted lines in Figure 1F–H, labeled as ROI_2_), respectively. Constants *a* and *b* are used as weights from 0.1 to 1.1 which can be adjusted empirically and fine‐tuned over multiple runs. A case was chosen with sufficient $B_{1}^{+}$ coverage by considering CV and maximum/minimum $B_{1}^{+}$ and performing visual inspection for symmetry and reduced dropout. The power splitter configuration used in this case is outlined in Figure 2B.

To generate a second, more flexible RF shim case (Case 2), a new optimization was performed, this time allowing the algorithm to modify both the amplitudes and phases on each channel. The same cost function was used (Eq. 1). A case was chosen to further extend coverage into the bottom half of the head while still providing sufficient coverage throughout. The amplitudes were rounded to the nearest implementable values. The splitter configuration used in this case is outlined in Figure 2C.

A third RF shim case (Case 3) was optimized using the same splitter configuration as in Case 2 (i.e., using the optimized amplitudes from Case 2) and performing phase‐only shimming, but utilizing a multi‐ROI approach, isolating regions where $B_{1}^{+}$ could be improved. In addition to the same top and bottom halves of the head models used in cases 1 and 2, two additional ROIs were placed: a slab around the axial center of the brain on all three models, and a small ROI around the left temporal lobe on the Duke model, which was chosen to isolate a commonly difficult‐to‐excite region at 7T. These ROIs are displayed in Figure 1E–H and labeled as ROI_3_ and ROI_4_. Additionally, a small ROI was placed around the center of the brain in the Duke model (ROI_5_, Figure 1F), where the maximum $B_{1}^{+}$ typically occurs; the mean in this ROI was constrained below 270° per 548 V to help keep the maximum values lower. The cost function used was

(2) $$\begin{array}{c} \text{Cost}=\frac{{\mathrm{CV}}_{\text{Duke}}}{a*\min_{1}+b*\min_{2}+c*\min_{3}+d*{\text{mean}}_{4}}\;\\ \text{Constraints}=\text{mean}>180{}^{\circ}/548V;\mathrm{mea}n_{5}<270{}^{\circ}/548V \end{array}$$

where *CV*_Duke_, *min*_*1*_, and *min*_*2*_ are as described above in Eq. 1, *min*_*3*_ is the minimum $B_{1}^{+}$ in the left temporal lobe of the Duke model, *mean*_*4*_ is the mean $B_{1}^{+}$ in the central slab, and *a*, *b*, *c*, and *d* are constants used for weighting as in Eq. 1.

### 2.4 SAR calculation

After optimization, SAR is calculated in MATLAB using the full resolution electric fields generated by FDTD and averaged per 10 g of tissue using the algorithm described by Caputa et al.^43^ SAR statistics are calculated over the whole head, from below the chin, as designated by the solid blue lines in Figure 1F–H. This ROI is chosen to give a conservative—worst case scenario—estimate of the average SAR.

### 2.5 Implementation

The coil was constructed using a 3D printed housing (ABS and polycarbonate plastic) with 17.5 μm‐thick copper shielding. 6.35 × 6.35 mm^2^ copper rods were inserted into each leg of the TTT structure on each panel. A 32‐channel receive insert was 3D printed to fit within the transmit coil.^44^ Each channel was tuned to 297.2 MHz and matched to 50 Ω using these tuning rods as well as fixed capacitors (Figure 1C) with the receive coil inside. The coupling between opposite ports on a single panel was −1.47 dB, and between adjacent ports on the same panel was −13.72 dB. The coupling panel‐to‐panel was no more than −18.00 dB. Decoupling circuits are attached to each TTT transmit panel. Each of the chosen optimal RF shim cases (Figure 2) was implemented so that the coil can be used in sTx mode on a Siemens 7T scanner. Wilkinson power splitters inspired by Yan et al.^45^ were built (Figure 2A), removing the adjustable power ratio circuit and expanding the design to achieve 2‐way through 8‐way configurations. Coaxial cables were used to shift the phase according to the optimization output. Losses are measured for each channel from the coil plug to the end of the cable that attaches to the coil. The average losses for the channels originating from each splitter are shown in Figure 2B,C.

### 2.6 Images acquired

$B_{1}^{+}$ maps were acquired on a MAGNETOM 7T scanner (Siemens) in sTx mode using a 3D Turbo‐FLASH sequence^46^ with the following parameters: TR/TE = 2000/1.16 ms; TA = 12 min; flip angle from 0° to 90° in 18° increments; and 3.2 mm isotropic resolution. The signal is fitted to a cosine function to calculate the estimated $B_{1}^{+}$ maps. $B_{1}^{+}$ maps were acquired on 17 healthy volunteers with informed consent as part of an approved study by the local institutional review board. The average age of the volunteers was 27.0 ± 2.8; nine were male and eight were female. All procedures complied with relevant guidelines and regulations for investigational use of the device in humans. For local SAR monitoring, the worst‐case SAR values were used for each implemented RF shim case, plus a safety margin of >2.

T_2_‐fluid attenuated inversion recovery (FLAIR) sequences were acquired on Volunteer 1 using RF shim cases 1 and 2, with the following parameters: TE/TI/TR = 99/2900/14000 ms, resolution 0.85 × 0.85 × 1.7 mm^3^, acceleration factor 2, 5 interleaved acquisitions (64 transversal slices), TA = 8:12 min. T_2_‐FLAIR sequences were acquired on Volunteer 2 using RF shim cases 2 and 3, with the following parameters: TE/TI/TR = 99/2900/14000 ms, resolution 0.75 × 0.75 × 1.5 mm^3^, acceleration factor 2, 7 interleaved acquisitions for Case 2 (80 transversal slices), 8 for Case 3 (100 transversal slices), TA = 11:28 min for Case 2, 13:06 min for Case 3.

## 3 RESULTS

**FIGURE 3:**
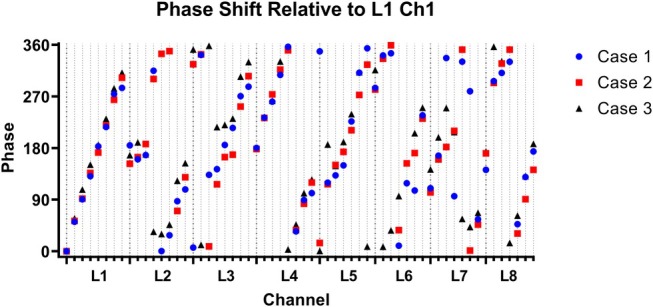
Plot of individual phases per channel for each RF shim case, sorted from channel 1–8 or 1–7 within each Z‐level (L1–L8) as outlined in Figure 1.

The final optimized phases for each of the three RF shim cases are shown in Figure 3. Individual channel phases are plotted, separated by Z‐level, and following the quadrature order of the channels in each Z‐level. The respective amplitudes can be determined from the splitter configurations in Figure 2.

Statistical values of the simulated $B_{1}^{+}$ field and SAR are listed in Table 1 for the three RF shim cases. Case 1 provides the highest mean $B_{1}^{+}$ field in the brain mask for all three head models; Case 3 provides the lowest coefficient of variation and maximum/minimum $B_{1}^{+}$ field in the brain mask for each model. Case 1 has the highest average SAR efficiency and Case 2 has the highest peak SAR efficiency, while Case 3 has the lowest average SAR for 1 W input power. Losses are included in the simulation results based on bench measurements of cables, splitters, and the plug and estimated loss in the transmit and receive coil and their components, including the TTT panels, ports, decoupling circuits, and losses in the dielectric and copper materials.^29^ As seen in Figure 2, bench measurements of the losses due to the plug, cables, and splitters range from 13.1% to 17.2%. Total losses ranged between 30% and 35% in all cases.

**TABLE 1:** Statistics of simulated $B_{1}^{+}$ field and SAR for three RF shim cases on three anatomically detailed head models. The same statistics are shown for the TEM coil on the Duke model.

	Mean $B_{1}^{+}$ (μT) for 1 W input	Mean flip angle (°/548 V) for 1 ms pulse width including RF losses after the coil socket	CV $B_{1}^{+}$	Max/min $B_{1}^{+}$	Avg SAR efficiency ($B_{1}^{+}$/√SAR_avg_)	Peak SAR efficiency ($B_{1}^{+}$/√SAR_peak_)	Avg SAR (W/kg) for 1 W input	Peak SAR (W/kg) for 1 W input	Peak/avg SAR
Case 1									
Duke	0.348	**209.57**	0.231	4.42	**1.55**	0.867	0.051	0.161	3.19
Ella	0.330	198.32	0.217	4.32	1.43	0.867	0.053	0.144	2.74
Billie	0.344	207.02	0.219	3.51	1.40	0.860	0.060	0.160	2.66
Case 2									
Duke	**0.373**	203.64	0.210	4.58	1.52	0.857	0.060	0.189	3.17
Ella	0.349	190.40	0.216	4.43	1.40	**0.873**	0.062	0.159	**2.59**
Billie	0.358	195.54	0.220	3.58	1.36	0.776	0.069	0.213	3.08
Case 3									
Duke	0.324	184.55	**0.197**	4.06	1.54	0.790	**0.044**	0.169	3.80
Ella	0.308	175.61	0.198	3.92	1.42	0.825	0.047	**0.140**	2.97
Billie	0.317	180.75	0.204	**3.49**	1.37	0.774	0.054	0.171	3.15
TEM Duke	0.403	N/A	0.314	294.25	1.16	0.600	0.121	0.450	3.73

*Note*: $B_{1}^{+}$ field statistics are calculated over each brain mask. The losses for the second column include losses from each case's splitter configuration (plug, cables, boards) and from the transmit and receive coils and their components. 548 V is the maximum output for our 7 T Siemens MAGNETOM (8 kW power amplifier). SAR statistics are calculated over the whole head above the chin. Bold numbers indicate best performance in each column.

Abbreviations: Avg, average; CV, coefficient of variation; Max, maximum; min, minimum; SAR, specific absorption rate; TEM, transverse electromagnetic.

Table 1 also provides summary statistics for the circularly polarized transverse electromagnetic (TEM) resonator as previously simulated on the Duke model using the same FDTD environment.^32^ The current study uses a larger brain mask that extends further down in the head‐foot direction than in the original reference; thus, the larger ROI was applied to the original TEM simulations. Despite a higher mean $B_{1}^{+}$, the peak and average SAR are higher with the TEM coil, resulting in lower peak and average SAR efficiency. Furthermore, the TEM coil provides a higher CV and maximum/minimum $B_{1}^{+}$.

**FIGURE 4:**
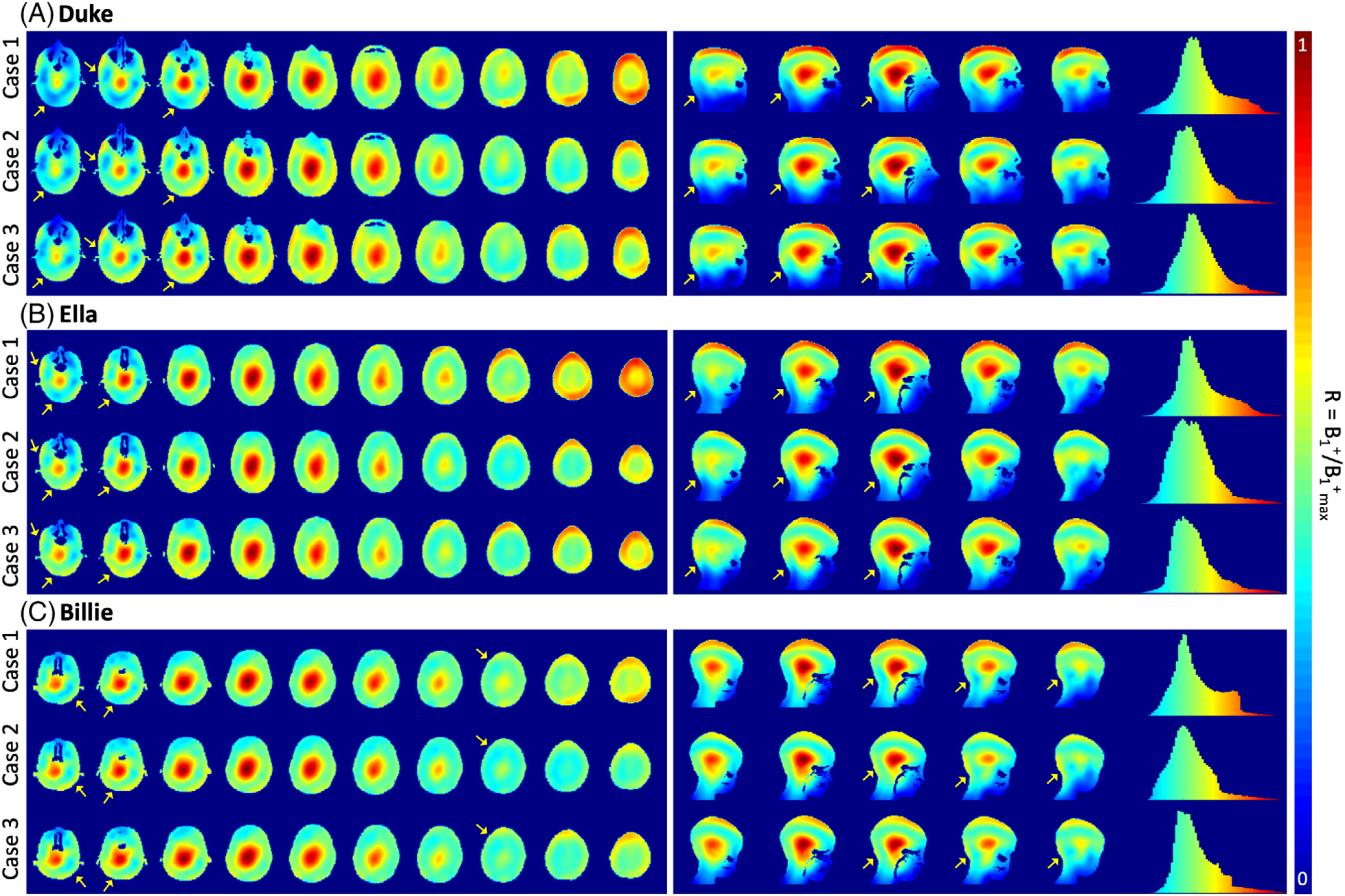
Simulated $B_{1}^{+}$ maps in three head models using three RF shim cases: (A) Duke model, cases 1, 2, and 3 from top row down; (B) Ella model, cases 1, 2, and 3; and (C) Billie model, cases 1, 2, and 3. For each model/case, the $B_{1}^{+}$ map is scaled as a ratio, *R*, from zero to one, where ${R=B}_{1}^{+}/B_{1}^{+}\max$, and $B_{1}^{+}\max$ is the maximum $B_{1}^{+}$ for that model/case. Histograms demonstrate the $B_{1}^{+}$ field distribution for each model/case within the brain mask, from the top of the head to bottom of cerebellum.

**FIGURE 5:**
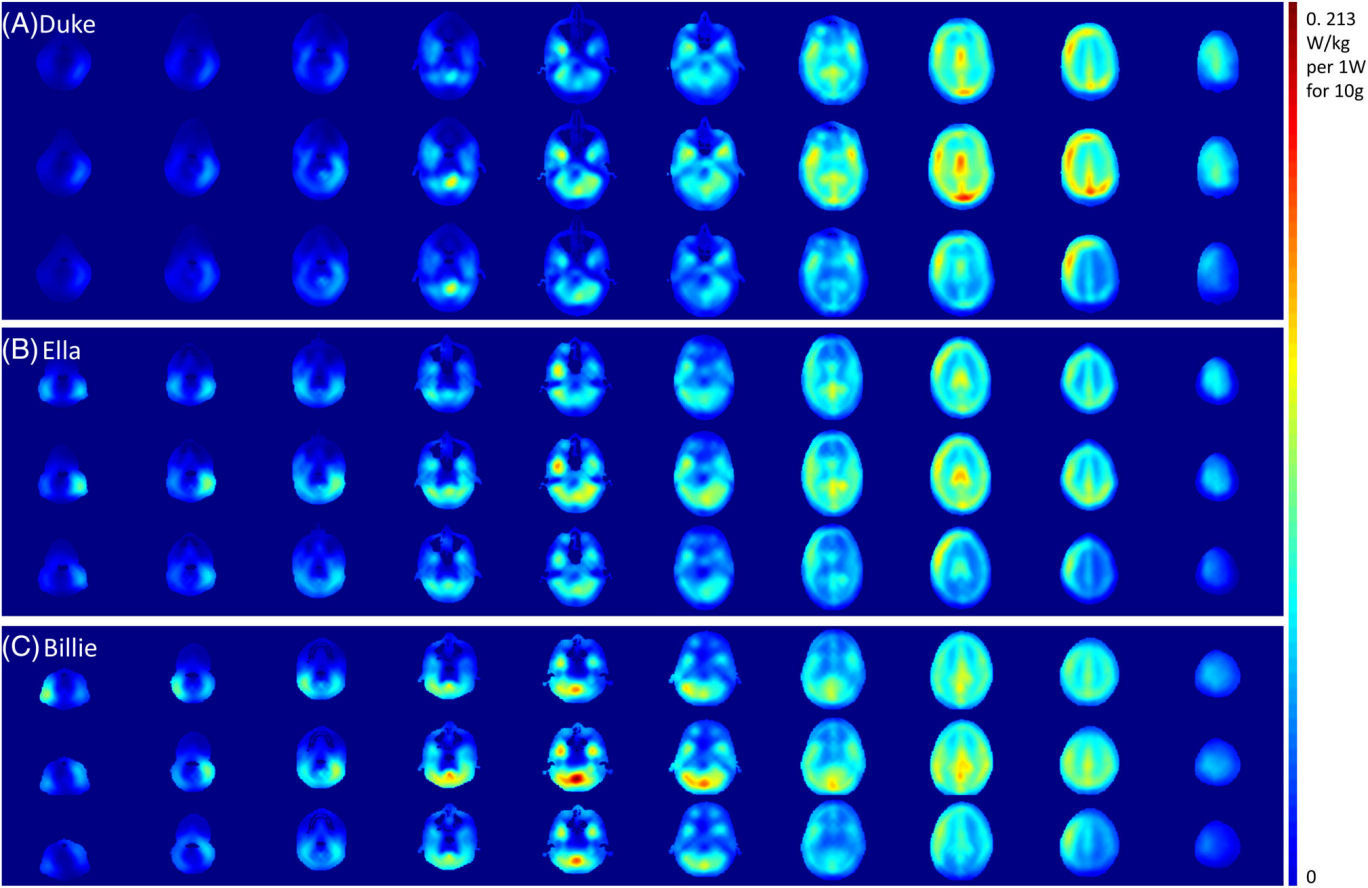
Simulated specific absorption rate (SAR) per 1 W in three head models using three RF shim cases, averaged over 10 g of tissue: (A) Duke model, cases 1, 2, and 3 from top row down; (B) Ella model, cases 1, 2, and 3; (C) Billie model, cases 1, 2, and 3. All cases/models are scaled to the same maximum value of 0.213 W/kg per 10 g of tissue (observed on the Billie model for Case 2) for 1 W input power.

Figure 4 shows the simulated $B_{1}^{+}$ maps on each of the three head models for each of the three finalized RF shim cases. Arrows highlight areas of improvement across the three shim cases, particularly in the cerebellum and left temporal lobe, and histograms are plotted of the $B_{1}^{+}$ field in each of the head masks. From Case 1 to Case 3, histograms become narrower with fewer low values. SAR maps for each case and each head model are displayed in Figure 5, shown in W/kg for 1 W input power, averaged over 10 g.

**FIGURE 6:**
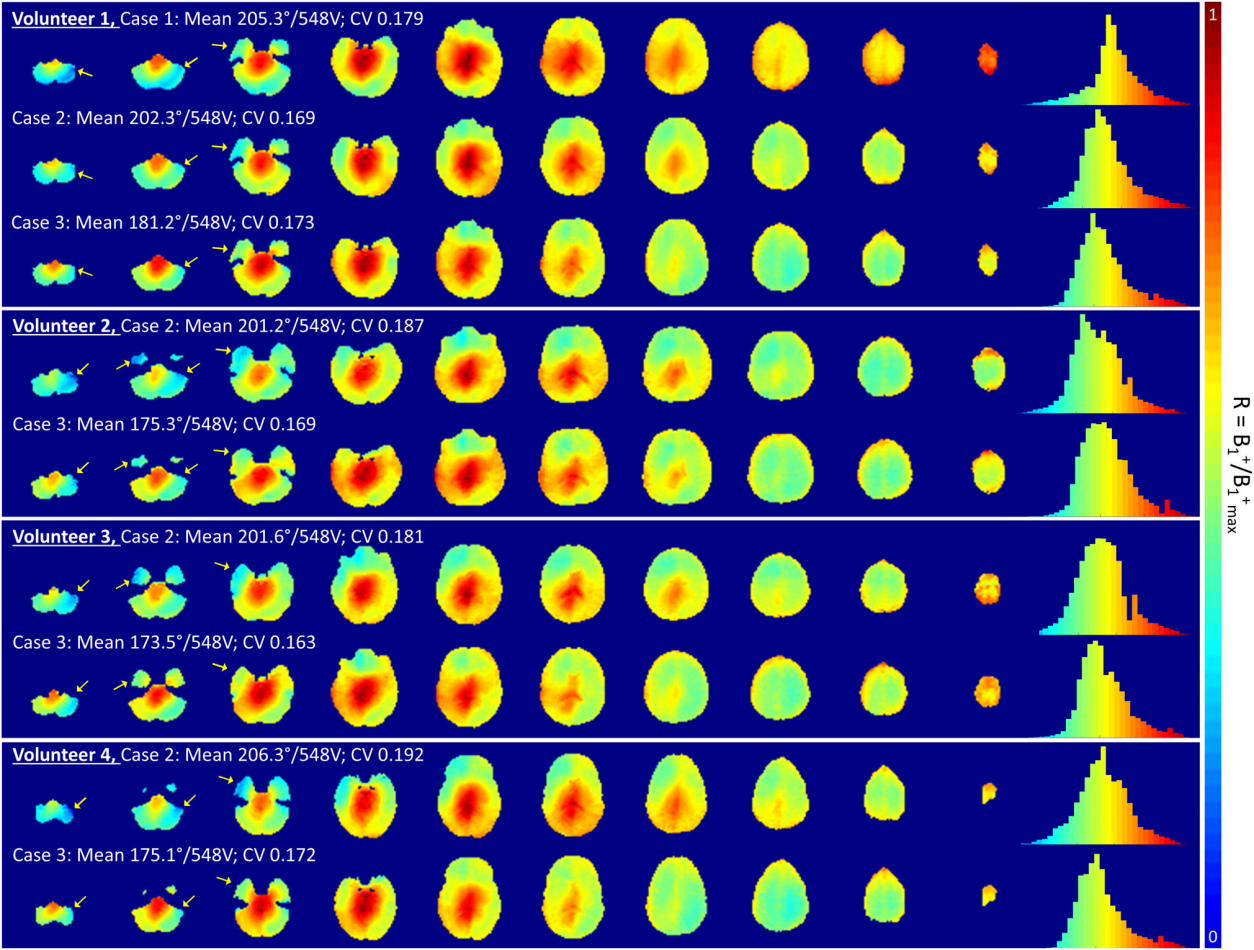
In vivo $B_{1}^{+}$ maps on volunteers who were scanned using the RF shim cases: one volunteer who was scanned using all three RF shim cases, and three volunteers using only Case 2 and Case 3. Each case/volunteer is scaled to its own maximum $B_{1}^{+}$, denoted as a ratio, R, from zero to one, where ${R=B}_{1}^{+}/B_{1}^{+}\max$. Histograms demonstrate the $B_{1}^{+}$ distribution across the brain for each volunteer/shim case, with the x‐axis going from zero to the maximum $B_{1}^{+}$ for that head model/case combination. The flip angle values assume 1 ms pulse width.

**FIGURE 7:**
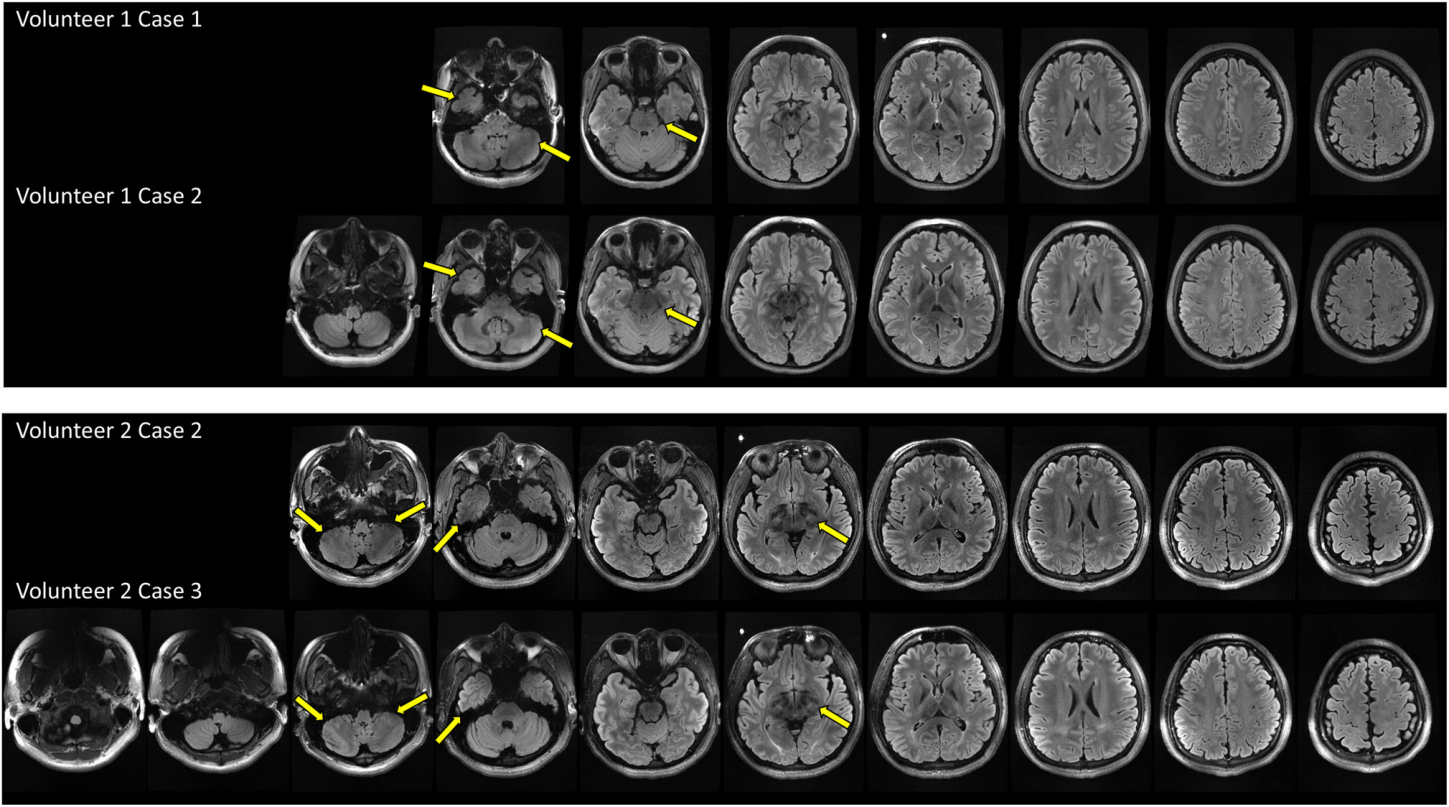
T_2_‐FLAIR images (raw from the scanner) acquired on volunteers 1 and 2 using the 60‐channel Tx/32‐channel Rx Tic Tac Toe RF coil system. The sequence used for Volunteer 1 was 64 slices, 0.85 × 0.85 × 1.7 mm resolution. The sequence used for Volunteer 2 was 0.75 × 0.75 × 1.5 mm resolution; Case 2 was 80 slices and Case 3 was 100 slices. Volunteer 1 has an intracranial volume (ICV) of 1.46 L, head dimensions of 19 cm AP, 16.2 cm RL, and 14.7 cm from the top of the skull to the bottom of the cerebellum. Volunteer 2 has an ICV of 1.67 L, head dimensions of 19.5 cm AP, 17.7 cm RL, and 15.2 cm from the top of the skull to the bottom of the cerebellum. Arrows show regions of improvement between the cases.

**FIGURE 8:**
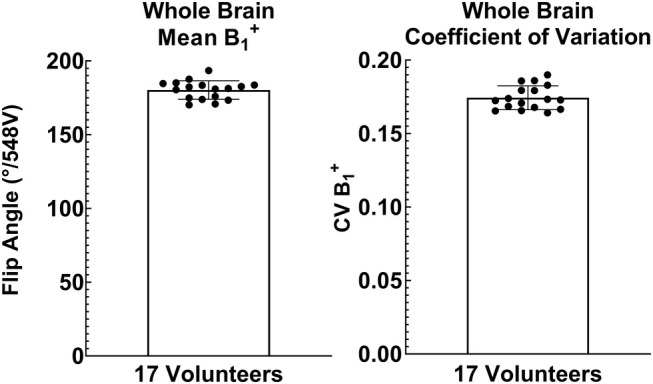
Measured $B_{1}^{+}$ statistics on 17 participants who were scanned with RF shim case 3. Plots of coefficient of variation (CV) and mean $B_{1}^{+}$ are shown with the bar representing the mean value and error bars for standard deviation. Mean $B_{1}^{+}$ is shown in terms of flip angle per 548 V for a 1 ms pulse width.

Figure 6 shows the $B_{1}^{+}$ field distribution in vivo in Volunteer 1, who was scanned with all three RF shimming cases, as well as three volunteers (2–4) who were scanned with RF shim Case 2 and Case 3. Volunteer age, sex, and ICV are as follows: volunteer 1, age 25, female, 1.46 L; volunteer 2, age 25, male, 1.67 L; volunteer 3, age 29, female, 1.57 L; and volunteer 4, age 26, female, 1.47 L. Figure 6 also shows histograms of $B_{1}^{+}$ values in the brain mask, and mean and CV values are also displayed for each volunteer. Mean $B_{1}^{+}$ decreases from Cases 1 and 2 to Case 3, but fewer lower values (typically observed in cerebellum and/or temporal lobes) are achieved where the CV also improves. Figure 7 shows FLAIR images (raw from the scanner) on Volunteer 1 (using Cases 1 and 2) and Volunteer 2 (using Cases 2 and 3). Arrows show regions of improvement between the cases. Figure 8 demonstrates the distribution of mean $B_{1}^{+}$ and CV across 17 volunteers (including Volunteers 1–4 from Figure 6) using Case 3 only. The average ICV was 1.54 ± 0.16 L. The average mean $B_{1}^{+}$ across the 17 volunteers was 180.25 ± 6.28° per 548 V for a 1 ms square pulse, and the average CV was 0.174 ± 0.0081.

## 4 DISCUSSION

In this work, we present RF shimming techniques for optimizing the 2nd Generation Tic Tac Toe RF coil system for routine use with the sTx systems at 7T. The increased number of transmit channels (60) improves efficiency in the azimuthal direction and provides more degrees of freedom (2*n* − 1 for *n* channels) for RF shimming as compared to the first generation Tic Tac Toe RF coil system with fewer transmit channels (16). To develop a robust and homogeneous implementation on the sTx system, three head models were used simultaneously, chosen to better reflect the demographics of the subjects scanned in ongoing studies at our facility. Optimizing over three head models of different sizes and positions enables the coil to perform well for a range of ages and head types while using the sTx mode of the scanner. The utilization of the sTx mode is the focus of this work, since this configuration does not require any complex and subject‐specific calibration in the scanner.

In addition to utilizing three head models in RF shimming, a multi‐ROI strategy was used. The first two shim cases developed in this work used a dual‐ROI strategy which aimed to improve $B_{1}^{+}$ field homogeneity in the lower regions of the brain (temporal lobe, brainstem, and cerebellum) that are often difficult to excite at 7T, regardless of coil design, while maintaining low CV across the head. The third shim case used several additional ROIs to isolate regions that should be further improved, increasing signal in the temporal lobe and improving homogeneity across the central parts of the brain. The ROIs used may be specific to our coil, but these are regions that are often problematic in other coil designs at 7T due to the wavelength and dielectric artifacts. Therefore, the same strategy can be applied to the optimization of any RF transmit array with small adjustments to the locations of the ROIs. Figure 4 highlights the improvements in simulation between shim cases 2 and 3, demonstrating the benefits of a multi‐ROI shimming strategy. These improvements are reflected in the in vivo field maps and images shown in Figures 5 and 6. This shimming framework is flexible to changing or adding ROIs and/or terms in the cost function, allowing for adjustments with coil geometry changes, and regions of too‐low or too‐high $B_{1}^{+}$ field can be specifically targeted and improved.

Furthermore, the power splitting strategy can largely impact $B_{1}^{+}$ and SAR. Case 1 and Case 2 used the same shimming method (i.e., the same cost function and ROIs) while using different power splitter configurations. As Case 1 was the earliest implementation of the coil, we fixed the amplitudes according to Z‐level, assigning more power to levels which had larger impact on overall $B_{1}^{+}$ field. For Case 2, by allowing free amplitude‐and‐phase shimming, even using the same cost function, the $B_{1}^{+}$ field distribution significantly improved. Case 3 uses the multi‐ROI cost function, but uses phase‐only shimming so that we could reuse the splitters implemented in Case 2. Further improvements could be achieved using a multi‐ROI shimming strategy and allowing for phases and amplitudes to vary.

The RF shimming cases developed in this work were implementable on the coil by using power splitters and coaxial cables of different lengths. The splitter configurations are shown in Figure 2. Case 1 uses a total of 11 power splitters, while the configuration used by both cases 2 and 3 requires 16 splitters. Despite the difference in the number of splitters used, the losses between cases do not vary significantly since the majority of channels are routed through 2 or 3 splitters in both configurations. Figure 2A displays some of the physical splitters used with the coil. They are able to be securely mounted behind the coil.

In vivo $B_{1}^{+}$ maps (Figure 6) demonstrate the improvements in homogeneity and extended coverage seen with cases 2 and 3. Since Case 1 was only in use for a limited amount of time, only one volunteer had been scanned using all three cases. Three volunteers were scanned with Case 2 and Case 3. FLAIR images (Figure 7) similarly show the improvements in $B_{1}^{+}$ field across the three cases. Some darkening in the FLAIR images can be seen in the middle of the brain due to over flipping, primarily in Case 2, which has the highest maximum/minimum $B_{1}^{+}$. Case 3 not only provides the lowest maximum/minimum $B_{1}^{+}$, but also allows us to achieve improved coverage below the cerebellum. $B_{1}^{+}$ maps were acquired on 17 volunteers with Case 3; the mean and CV of $B_{1}^{+}$, shown in Figure 8, present low variability (≤7.3% and ≤8.9%, respectively) across subjects.

In order to achieve improved $B_{1}^{+}$ field homogeneity in Case 3, some tradeoffs must be made in overall power efficiency. Nonetheless, the coil is still able to provide sufficient $B_{1}^{+}$ field intensity with an average flip angle from simulations and experiments of ˜180° with 1 ms pulse width at the scanner's allowed voltage. Additionally, the ratio of peak to average SAR is highest in Case 3, demonstrating another tradeoff to achieve homogeneous $B_{1}^{+}$ field, although the SAR levels are still relatively low.^32^ In this work, we did not include a SAR parameter in our optimization cost function. The addition of a SAR parameter in the cost function could allow the optimization software to balance homogeneity and SAR.^30^, ^47^ Even without the inclusion of the SAR in the cost function, safe levels of SAR are achieved across all models and in all three cases presented here. In the in vivo data acquired, the worst‐case predicted head local SAR was 1.97 W/kg, much lower than the 10 W/kg IEC local SAR limit. Ultimately, this design and optimization strategy is uniquely flexible in allowing for tradeoffs in power efficiency, homogeneity, and SAR. An ideal Tic Tac Toe design would benefit from additional power, beyond the 8 kW power amplifiers at our scanner which reduces to ˜6 kW at the coil plug.

To examine how the 2nd generation Tic Tac Toe RF coil system compares to more standard birdcage designs, a TEM resonator can be used. Previous work^32^ used the same FDTD simulation environment as described here on the Duke model, therefore a fair comparison can be made by calculating the $B_{1}^{+}$ measures over the larger ROI used in this work. The TEM is more power efficient (i.e., able to provide higher $B_{1}^{+}$ on average) but does not perform as well as the TiTT in terms of peak or average SAR efficiency. Further, the TEM provides less homogeneous $B_{1}^{+}$, with a CV of 0.314. The very high maximum/minimum $B_{1}^{+}$ observed with the TEM coil is partially attributed to the extended ROI used in this work, which now includes regions in the lower brain with very low $B_{1}^{+}$ intensities. In the literature, the standard commercial sTx coil is reported to have in vivo $B_{1}^{+}$ CV of 0.22 to 0.24 without the use of dielectric pads,^48^, ^49^ and 0.20 to 0.27 with the use of dielectric pads;^48^, ^49^, ^50^ in each study, CV is reported for 5 to 6 subjects. The 2nd generation Tic Tac Toe RF coil system performs well in comparison, with all in vivo CV values <0.2 (ranging from 0.163–0.192 across all subjects and all RF shim cases) without the need for dielectric pads.

The 2nd generation Tic Tac Toe RF coil system is used on a regular basis to acquire a range of contrasts and sequences: structural (T_1_‐weighted, T_2_‐weighted, FLAIR), functional, diffusion, perfusion, angiography, spectroscopy, magnetization transfer, and QSM tailored gradient echo. Current studies are being conducted using RF shim case 3. To date, this coil has been used in 36 active or concluded studies (33 National Institutes of Health‐funded, 3 other sources) with >1750 in vivo human scan sessions in 2.5 years, many of which include sequences that are difficult to acquire at 7T such as T_2_w‐FLAIR, T_2_w‐SPACE, T_2_w‐TSE, pCASL, and SS‐diffusion MRI, supporting the mitigation of $B_{1}^{+}$ inhomogeneities and the availability of sufficient power efficiency.

Limitations of this work include the absence of the receive coil in the simulation and its experimental evaluation, as well as the absence of $B_{1}^{+}$ maps with and without the receive coil or individual‐channel $B_{1}^{+}$ maps. Additionally, although we reported other works that compare to the standard commercial sTx coils, this work lacks direct comparison using the same simulation environment and/or the same acquisition parameters. Future work will include such comparisons.

## 5 CONCLUSION

The presented RF shimming techniques implemented on the 2nd generation Tic Tac Toe RF coil system demonstrate the ability to provide non‐subject‐specific homogeneous $B_{1}^{+}$ field excitation for routine neuroimaging at 7T MRI on the sTx mode. The 60‐channel design improves power efficiency and enables a higher degree of RF shimming flexibility compared to the original 16‐channel TTT coil, achieving extended brain coverage while maintaining patient comfort with an open visual field. Three optimized non‐subject specific RF shim cases, developed using three head models with a wide range of head sizes from the IT'IS Foundation Virtual Family,^42^ exhibit improvements in homogeneity and coverage, particularly in challenging‐to‐image regions such as the temporal lobes and cerebellum at 7T. These RF shim cases also show safe SAR levels across all models, making this coil system an effective tool for routine 7T neuroimaging, already with >1750 in vivo human scanning sessions over the past 28 months.
